# Supplementary material for: Burden of Influenza and Respiratory Syncytial Viruses in Suspected COVID-19 Patients: A Cross-Sectional and Meta-Analysis Study
Source: Viruses. 2023 Mar 1;15(3):665. doi: 10.3390/v15030665 (PMC10055802; doi:10.3390/v15030665)

File S1

Table S1. PRISMA 2009 checklist.

Section/topic	#	Checklist item	Reported on page #
**TITLE**			
Title	1	Identify the report as a systematic review, meta-analysis, or both.	01
**ABSTRACT**			
Structured summary	2	Provide a structured summary including, as applicable: background; objectives; data sources; study eligibility criteria, participants, and interventions; study appraisal and synthesis methods; results; limitations; conclusions and implications of key findings; systematic review registration number.	01
**INTRODUCTION**			
Rationale	3	Describe the rationale for the review in the context of what is already known.	02-03
Objectives	4	Provide an explicit statement of questions being addressed with reference to participants, interventions, comparisons, outcomes, and study design (PICOS).	04-05
**METHODS**			
Protocol and registration	5	Indicate if a review protocol exists, if and where it can be accessed (e.g., Web address), and, if available, provide registration information including registration number.	04
Eligibility criteria	6	Specify study characteristics (e.g., PICOS, length of follow-up) and report characteristics (e.g., years considered, language, publication status) used as criteria for eligibility, giving rationale.	04-05
Information sources	7	Describe all information sources (e.g., databases with dates of coverage, contact with study authors to identify additional studies) in the search and date last searched.	04
Search	8	Present full electronic search strategy for at least one database, including any limits used, such that it could be repeated.	04
Study selection	9	State the process for selecting studies (i.e., screening, eligibility, included in systematic review, and, if applicable, included in the meta-analysis).	04
Data collection process	10	Describe method of data extraction from reports (e.g., piloted forms, independently, in duplicate) and any processes for obtaining and confirming data from investigators.	04-05
Data items	11	List and define all variables for which data were sought (e.g., PICOS, funding sources) and any assumptions and simplifications made.	04-05
Risk of bias in individual studies	12	Describe methods used for assessing risk of bias of individual studies (including specification of whether this was done at the study or outcome level), and how this information is to be used in any data synthesis.	04-05
Summary measures	13	State the principal summary measures (e.g., risk ratio, difference in means).	05

Synthesis of results	14	Describe the methods of handling data and combining results of studies, if done, including measures of consistency (e.g., I^2) for each meta-analysis.	05

Section/topic	#	Checklist item	Reported on page #
Risk of bias across studies	15	Specify any assessment of risk of bias that may affect the cumulative evidence (e.g., publication bias, selective reporting within studies).	04-05
Additional analyses	16	Describe methods of additional analyses (e.g., sensitivity or subgroup analyses, meta-regression), if done, indicating which were pre-specified.	04-05
**RESULTS**			
Study selection	17	Give numbers of studies screened, assessed for eligibility, and included in the review, with reasons for exclusions at each stage, ideally with a flow diagram.	05
Study characteristics	18	For each study, present characteristics for which data were extracted (e.g., study size, PICOS, follow-up period) and provide the citations.	05-06
Risk of bias within studies	19	Present data on risk of bias of each study and, if available, any outcome level assessment (see item 12).	04
Results of individual studies	20	For all outcomes considered (benefits or harms), present, for each study: (a) simple summary data for each intervention group (b) effect estimates and confidence intervals, ideally with a forest plot.	04-07
Synthesis of results	21	Present results of each meta-analysis done, including confidence intervals and measures of consistency.	04-08
Risk of bias across studies	22	Present results of any assessment of risk of bias across studies (see Item 15).	04,08
Additional analysis	23	Give results of additional analyses, if done (e.g., sensitivity or subgroup analyses, meta-regression [see Item 16]).	04-07
**DISCUSSION**			
Summary of evidence	24	Summarize the main findings including the strength of evidence for each main outcome; consider their relevance to key groups (e.g., healthcare providers, users, and policy makers).	09-10
Limitations	25	Discuss limitations at study and outcome level (e.g., risk of bias), and at review-level (e.g., incomplete retrieval of identified research, reporting bias).	09-10
Conclusions	26	Provide a general interpretation of the results in the context of other evidence, and implications for future research.	10
**FUNDING**			
Funding	27	Describe sources of funding for the systematic review and other support (e.g., supply of data); role of funders for the systematic review.	10

From: Moher D, Liberati A, Tetzlaff J, Altman DG, The PRISMA Group (2009). Preferred Reporting Items for Systematic Reviews and Meta-Analyses: The PRISMA Statement. PLoS Med 6(7): e1000097. doi:10.1371/journal.pmed1000097

Figure S1: PRISMA 2009 checklist showing guidelines for systematic reviews and meta-analysis.
For more information, visit: **www.prisma-statement.org**.

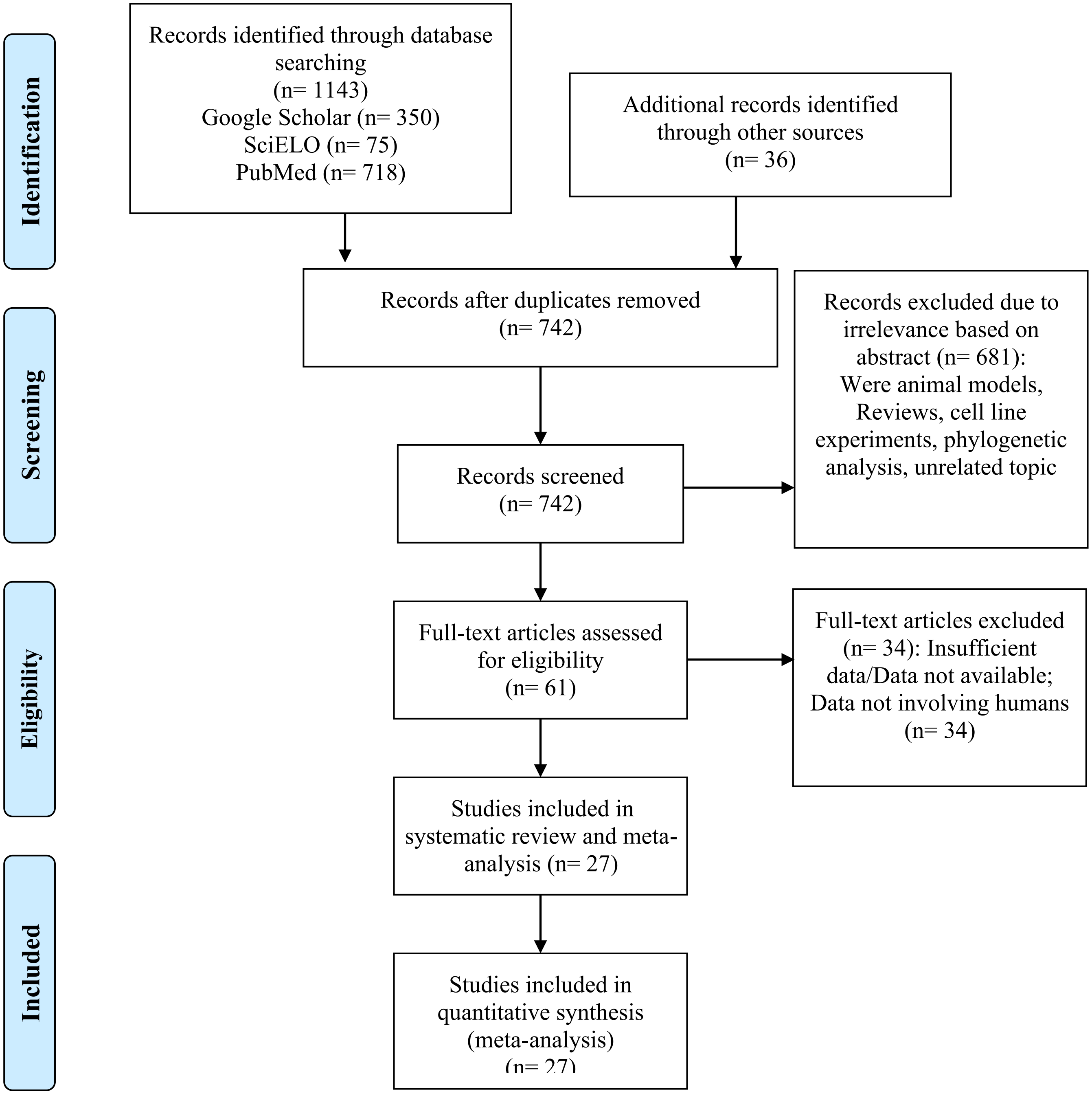

Table S2. The characteristics of the studies included in the systematic review and meta-analysis.

	ID[1]	Biological material collected	Diagnostic method	SARS-CoV negative						Gender (all patients)	Study period	Place of study
				FluAV POS	FluAV NEG	FluBV POS	FluBV NEG	RSV POS	RSV NEG			
1	Muruganandam, N. et al. 2022. Respiratory viruses among ethnic Nicobarese during COVID-19 pandemic. BMC Infec Dis.	Nasal/throat swabs	qPCR	51	173	0	0	0	224	Female (50.5%)	Mar-20-Abr-21	Car-Nicobar, India
2	Kriger, Or. et al. 2022. Viral viral co-pathogens in COVID-19 acute respiratory syndrome – what did we learn from the first year of pandemic? Int J Infec Dis.	Oro-nasopharyngeal swabs	Multiplex qPCR	6	457	0	476	0	476	Female (42.2%)	Mar-20 Feb-21	Ramat Gan, Israel
3	Schirmer, P. et al. 2021. Respiratory co-infections with COVID-19 in the Veterans Health Administration, 2020. Diagn Microbiol Infect Dis.	-	Multiplex qPCR	187	1022	54	1022	101	1022	Female (13.7%)	Fev-20 May-21	Puerto Rico, USA
4	Hirotsu, Y. et al. 2020. Analysis of Covid-19 and non-Covid-19 viruses, including influenza viruses, to determine the influence of intensive preventive measures in Japan. J Clin Virol.	Nasopharyngeal swabs	qPCR	0	183	0	183	0	183	-	Mar-20 May-20	Tokyo, Japan
5	Hsih, W.H. et al. 2020. Featuring COVID-19 cases via screening symptomatic patients with epidemiologic link during flu season in a medical center of central Taiwan. J Microbiol Immunol Infect.	Oro-nasopharyngeal swabs	Nested-qPCR	3	38	1	38	2	38	Female (60.4%)	Jan-20 Feb-20	Taichung, Taiwan
6	Stowe, J. et al. 2021. Interactions between SARS-CoV-2 and influenza, and the impact of coinfection on disease severity: a test-negative design. Int J Epidemiol.	-	PCR	992	14755	-	-	-	-	Female (49.3%)	Jan-20 Apr-20	England
7	Akhatar, Z. et al. 2021. SARS-CoV-2 and influenza virus coinfection among patients with severe acute respiratory infection during the first wave of COVID-19 pandemic in Bangladesh: a hospital-based descriptive study. BMJ Open.	Oro-nasopharyngeal swabs	qPCR	175 (FluV)	1986	-	-	-	-	Female (33.6%)	Mar-20 Dec-0	Bangladesh
8	Alpaydin, A.O. et al. 2020. Clinical and radiological diagnosis of non–SARS-CoV-2 viruses in the era of COVID-19 pandemic. J Med Virol.	Nasopharyngeal swabs	Multiplex qPCR	0	78	0	78	3	78	Female (48.71%)	Mar-20	Turkey
9	Babiker, A. et al. 2021. Metagenomic sequencing to detect respiratory viruses in persons under investigation for COVID-19. J Clin Microbiol.	Nasopharyngeal swabs	Metagenomic NGS	3	30	-	-	2	30	Female (51.6%)	Feb-20 Apr-20	Georgia, USA
10	Blasco, M.L. et al. 2020. Co-detection of respiratory pathogens in patients hospitalized with Coronavirus viral disease-2019 pneumonia. J Med Virol.	Oro-nasopharyngeal swabs	qRT-PCR	1	23	-	-	1	23	-	Mar-20	Valencia, Spain
11	Castillo, E.M. et al. 2020. Rates of coinfection with other respiratory pathogens in patients positive for coronavirus disease 2019 (COVID-19). JACEP Open.	Nasal/throat swabs	RPNA-RT-PCR	1	326	0	326	4	326	Female (50.65%)	Mar-20	San Diego, USA
12	Eisen, A.K.A. et al. 2021. Low circulation of Influenza A and coinfection with SARS-CoV-2 among other respiratory viruses during the COVID-19 pandemic in a region of southern Brazil. J Med Virol.	Oro-nasopharyngeal swabs	qRT-PCR	10	569	0	569	-	-	-	Mar-20 Dec-20	Rio Grande do Sul,
13	Freeman, C.L. et al. 2021. Co-detection of SARS-CoV-2 with secondary respiratory pathogen infections. J Gen Intern Med.	-	Multiplex qPCR	46	686	12	686	20	686	-	Mar-20 Oct-20	Nashville, USA
14	Hazra, A. et al. 2020. Coinfections with SARS-CoV-2 and other respiratory pathogens. Infect Control Hosp Epidemiol.	Nasopharyngeal swabs	qRT-PCT/RT-PCR-	49	2076	13	2076	39	2076	-	Mar-20 Apr-21	Chicago, USA
15	Kim, D. et al. 2020. Rates of co-infection between SARS-CoV-2 and other respiratory pathogens. JAMA.	Nasopharyngeal swabs	qRT-PCR	29	1101	8	1101	32	1101	Female (54.85%)	Mar-20	California
	Marshall, N.C. et al. 2021. Broad respiratory testing to identify	-	qRT-	555	51129	236	51129	438	51129	-	Mar-20	Edmonton,

16	SARS‑CoV‑2 viral co‑circulation and inform diagnostic stewardship in the COVID‑19 pandemic. Virol J.		PCR								May-20	Canada
17	Matos, A.R. et al. 2020. Identification of SARS-CoV-2 and additional respiratory pathogens cases under the investigation of COVID-19 initial phase in a Brazilian reference laboratory. Mem Inst Oswaldo Cruz.	Nasopharyngeal swabs	qRT-PCR	165	9	9	165	5	165	-	Feb-20 Mar-20	Brazil
18	Nowak, M.D. et al. 2020. Coinfection in SARS-CoV-2 infected patients: where are influenza virus and rhinovirus/enterovirus? J Med Virol.	-	Multiplex qRT-PCR	10	845	3	845	13	845	Female (53.96%)	Mar-20 Apr-20	New York, USA
19	Schneider, J.G. et al. 2021. Identifying risk factors that distinguish symptomatic severe acute respiratory syndrome coronavirus 2 infection from common upper respiratory infections in children. Cureus.	Nasopharyngeal swabs	qRT-PCR/RT-PCR	1	243	2	243	10	243	Female (48.16%)	Mar-20 May-20	Indianapolis, USA
20	Shah, S.J. et al. 2020. Clinical features, diagnostics, and outcomes of patients presenting with acute respiratory illness: A retrospective cohort study of patients with and without COVID-19. EClinicalMedicine.	Oro-nasopharyngeal swabs	RT-PCR	5	194	2	194	3	194	Female (49.46%)	Feb-20 Mar-20	San Francisco, USA
21	Singh, V. et al. 2021. SARS-CoV-2 respiratory co-infections: incidence of viral and bacterial co-pathogens. Int J Infect Dis.	Nasal/oropharyngeal, and sputum swabs	qRT-PCR	6	16363	-	-	16	16363	Female (55.2%)	Mar-20 Aug-20	Denton, USA
22	Chekuri, S. et al. 2021. SARS-CoV-2 coinfection with additional respiratory virus does not predict severe disease: a retrospective cohort study. J Antimicrob Chemother.	Nasopharyngeal swabs	Multiplex qRT-PCR	8	88	4	88	10	88	-	Mar-20 Apr-20	New York, USA
23	Letafati, A. et al. 2022. No human respiratory syncytial virus but SARS-CoV-2 found in children under 5 years old referred to children medical Center in 2021, Tehran, Iran. J Med Virol.	Nasopharyngeal swabs	qRT-PCR	-	-	-	-	0	152	Female (44%)	Mar-20 May-21	Tehran, Iran
24	Varela, F.H. et al. 2021. Absence of detection of RSV and influenza during the COVID-19 pandemic in a Brazilian cohort: Likely role of lower transmission in the community. J Glob Health.	Oro-nasopharyngeal swabs	qRT-PCR	0	966	0	966	0	966	Female (58.88%)	May-20 Aug-20	Porto Alegre, Brazil
25	Boschiero, M.N. et al. 2022. Frequency of respiratory pathogens other than SARS-CoV-2 detected during COVID-19 testing. Diagn Microbiol Infect Dis.	-	qRT-PCR	113	2792	182	4252	90	2810	-	2020	São Paulo, Brazil
26	Zhang, D.D. et al. 2020. Characterizing coinfection in children with COVID-19: A dual center retrospective analysis. Infect Control Hosp Epidemiol.	-	Multiplex qRT-PCR	4	666	12	666	25	666	-	Mar-20 Apr-20	Chicago, USA
27	Chung, H.Y. et al. 2021. Novel dual multiplex real-time RT-PCR assays for the rapid detection of SARSCoV-2, influenza A/B, and respiratory syncytial virus using the BD MAX open system. Emerg Infect Dis.	Nasopharyngeal swabs	Multiplex qRT-PCR	19	150	5	150	2	150	Female (49.26%)	Feb-20 Aug-20	Taipei, Taiwan

[1]ID: Identification of the study;

Table S3. Methodologic quality of cohort and case-control studies included in the meta-analysis.

Methodologic quality of cohort studies included in the meta-analysis (NOS)

The “star” presents a “high-quality” choice of individual study. For high-quality study was defined as a study with ≥4 awarded stars.

Methodologic quality of case-control studies included in the meta-analysis (Newcastle-Ottawa scale (NOS))

Study and year	Representa-tiveness of cases	Selection of non-exposed	Ascertainment of exposure	Demonstration outcome	Assessment of outcome	Follow-up outcomes	Adequacy of follow-up	Total quality scores
Muruganandam, N. et al. 2022	☆	☆	-	☆	☆	-	-	4
Kriger, Or. et al. 2022	☆	☆		☆	☆		-	4
Schirmer, P. et al. 2021	☆	☆	-	☆	☆	-	-	4
Hirotsu, Y. et al. 2020	☆	☆	-	☆	☆	-	-	4
Hsih, W.H. et al. 2020	☆	☆	–	☆	☆		-	4
Stowe, J. et al. 2021	☆	☆	-	☆	☆	-	-	4
Akhatar, Z. et al. 2021	☆	☆	-	☆	☆	-	-	4
Alpaydin, A.O. et al. 2020	☆	☆	-	☆	☆	-	-	4
Babiker, A. et al. 2021	☆	☆	–	☆	☆		-	4
Blasco, M.L. et al. 2020	☆	☆	-	☆	☆	-	-	4
Castillo, E.M. et al. 2020	☆	☆	-	☆	☆	-	-	4
Eisen, A.K.A. et al. 2021	☆	☆	-	☆	☆	-	-	4
Freeman, C.L. et al. 2021	☆	☆	-	☆	☆	-	-	4
Hazra, A. et al. 2020	☆	☆	-	☆	☆	-	-	4
Kim, D. et al. 2020	☆	☆	-	☆	☆	-	-	4
Marshall, N.C. et al. 2021	☆	☆		☆	☆		-	4
Matos, A.R. et al. 2020	☆	☆	-	☆	☆	-	-	4
Nowak, M.D. et al. 2020	☆	☆	–	☆	☆	-	-	4
Schneider, J.G. et al. 2021	☆	☆	-	☆	☆		-	4
Shah, S.J. et al. 2020	☆	☆	-	☆	☆	-	-	4
Singh, V. et al. 2021	☆	☆	–	☆	☆	-	-	4
Chekuri, S. et al. 2021	☆	☆	-	☆	☆	-	-	4
Letafati, A. et al. 2022	☆	☆	-	☆	☆	-	-	4
Varela, F.H. et al. 2021	☆	☆	-	☆	☆	-	-	4
Boschiero, M.N. et al. 2022	☆	☆	-	☆	☆	-	-	4
Zhang, D.D. et al. 2020	☆	☆	-	☆	☆	-	-	4
Chung, H.Y. et al. 2021	☆	☆	-	☆	☆	-	-	4

Figure S2. Forest plot containing positivity according to FluV and RSV subtypes.

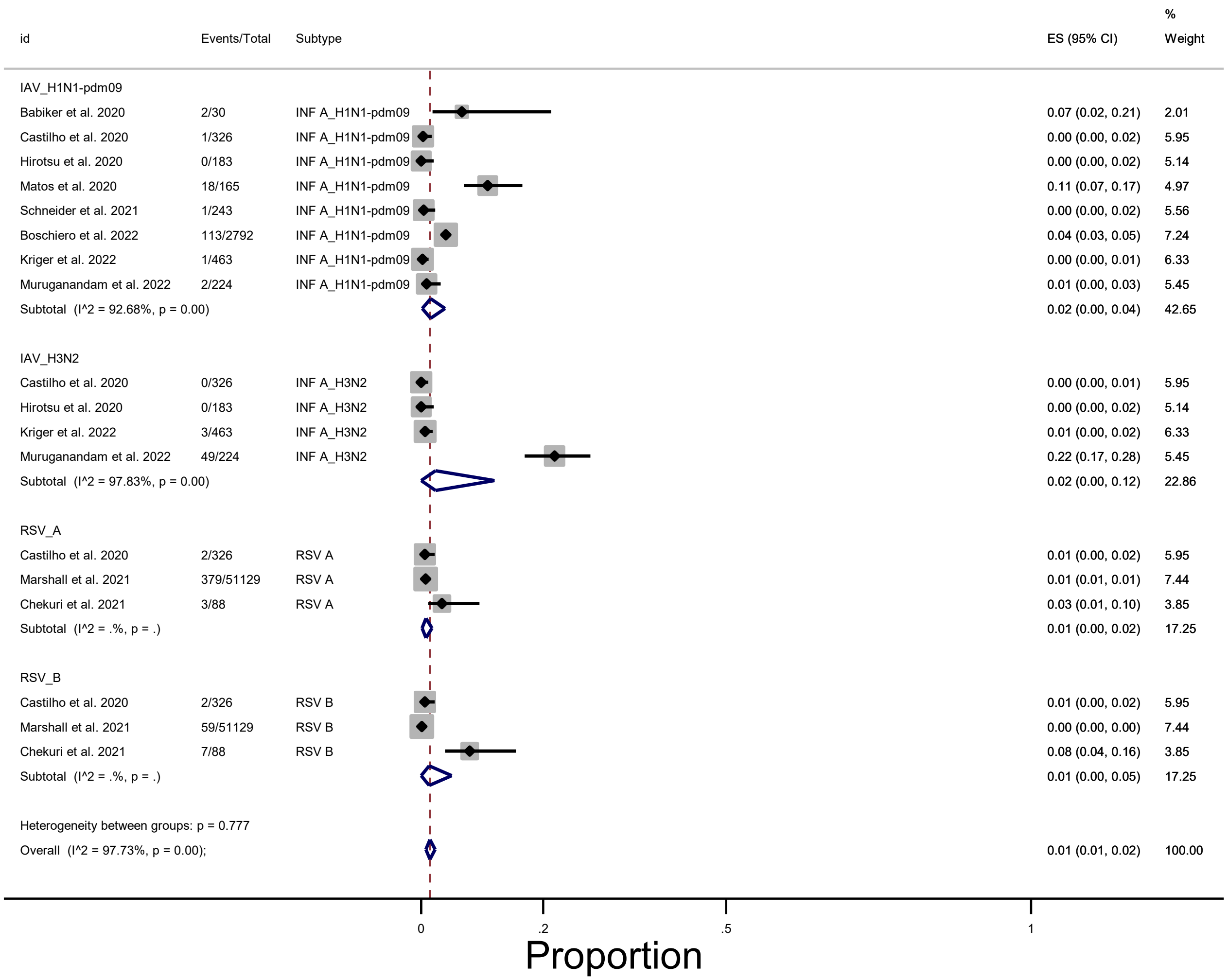

Figure S3. FluV in relation to the year of collection of biological samples.

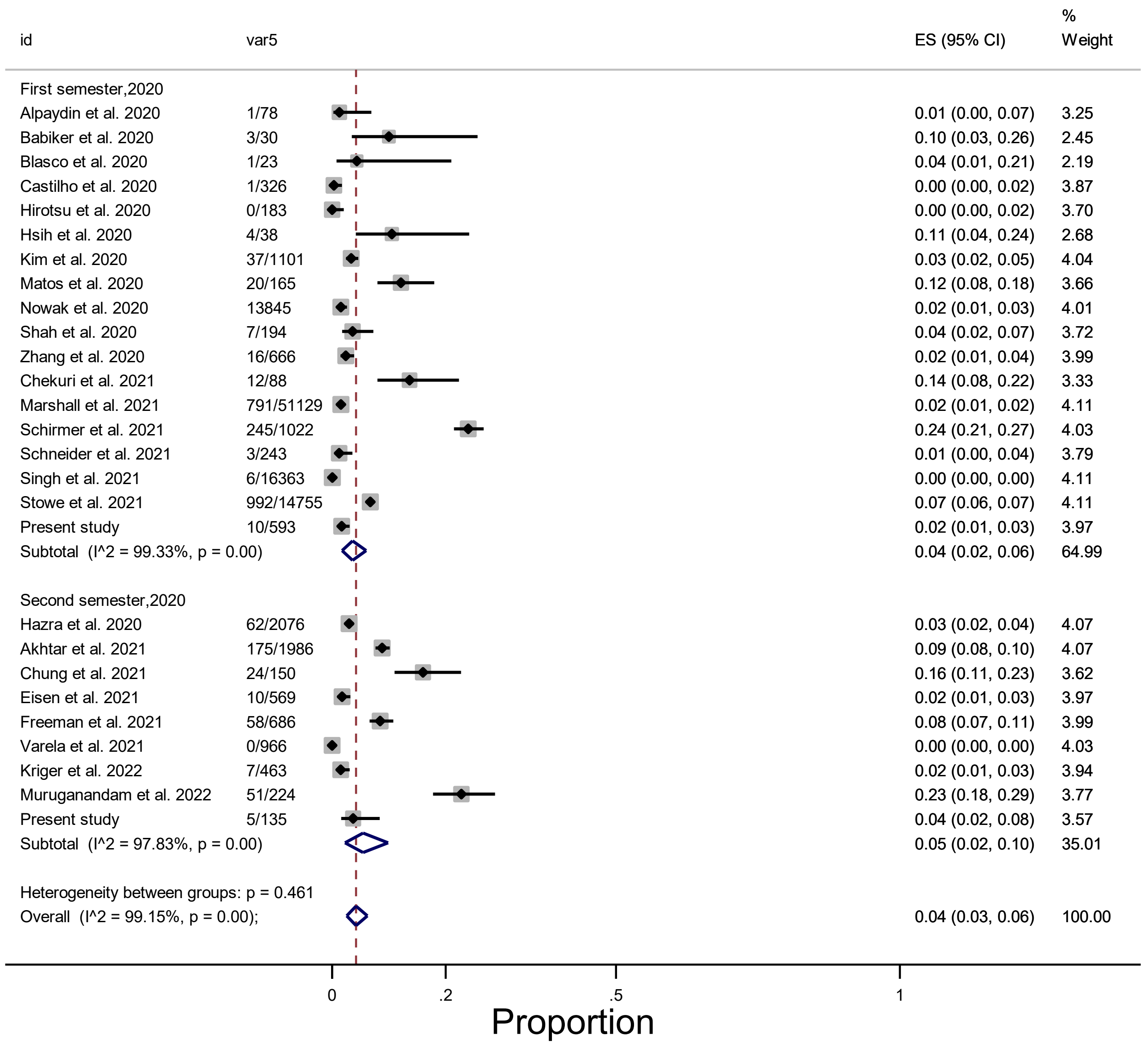

Figure S4. RSV positivity in relation to the year of collection of biological samples.

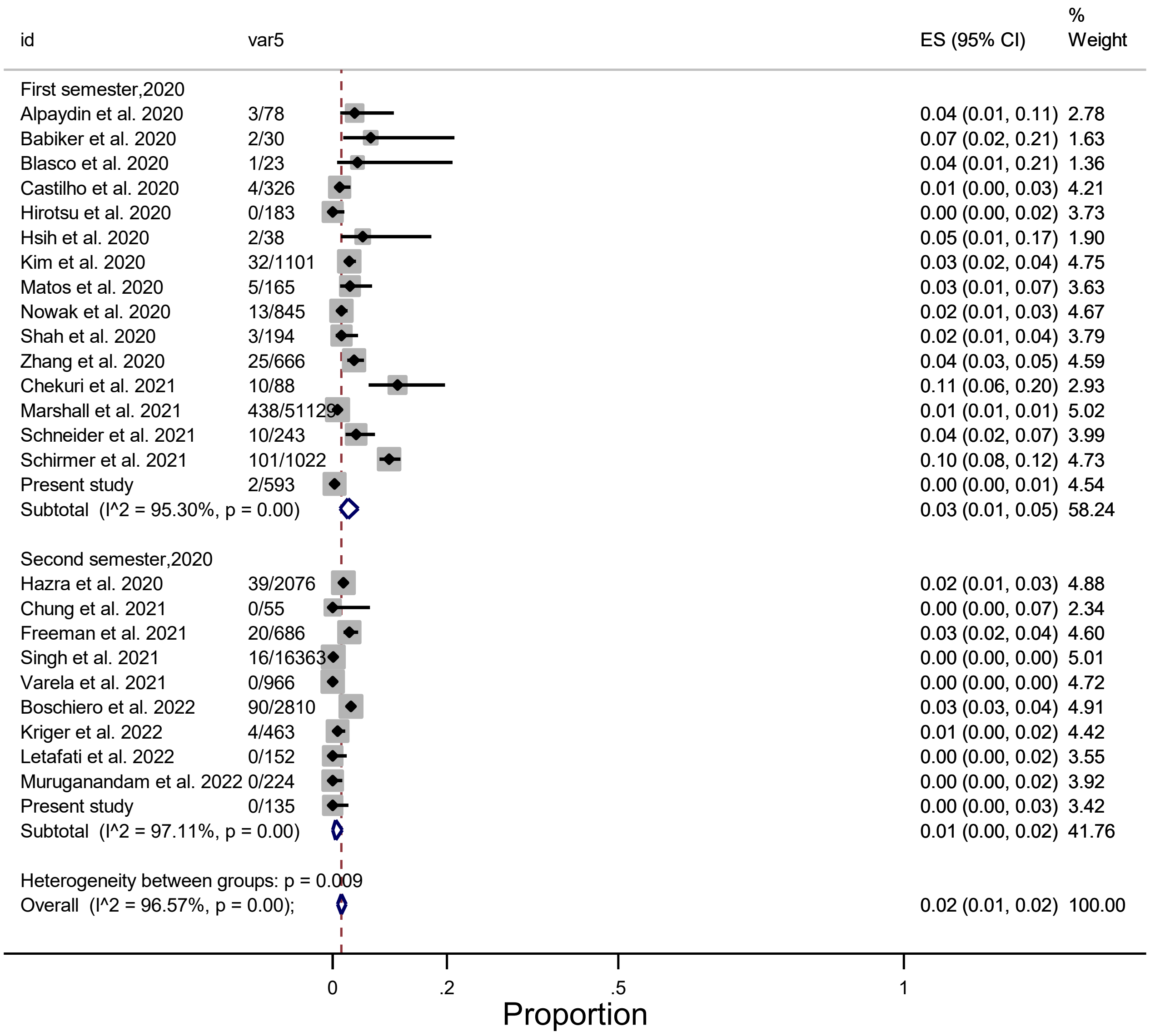

Figure S5. Funnel plot for FluV positivity among SCNG.

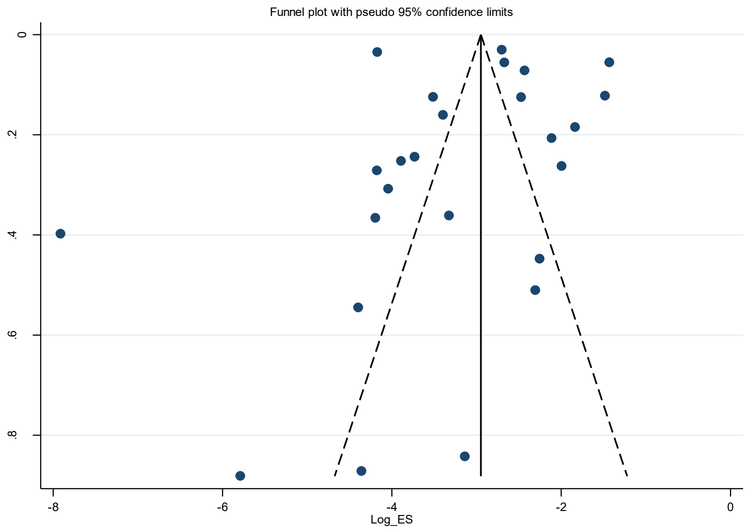

Figure S6. Funnel plot for FluAV positivity among SCNG

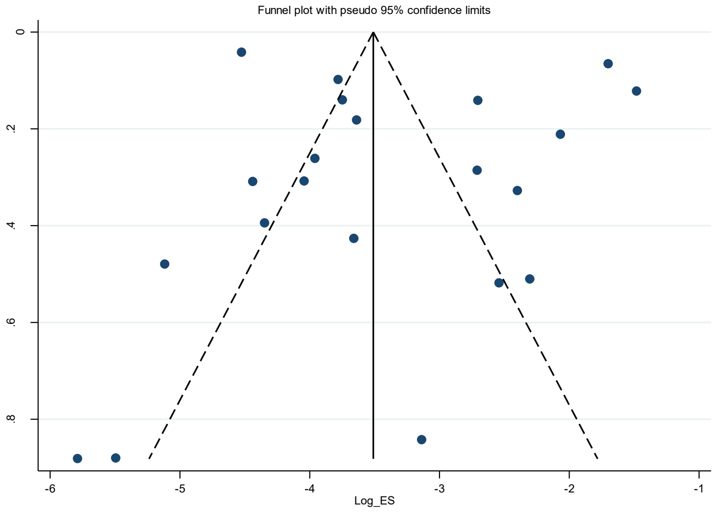

Figure S7. Funnel plot for FluBV positivity among SCNG.

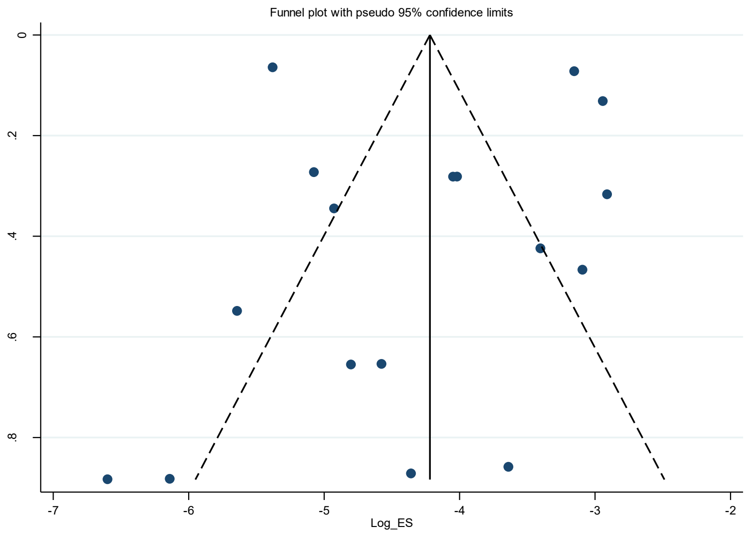

Figure S8. Sensitivity analysis for the effect of removing one study at a time on the overall estimation of FluV positivity in SCNG.

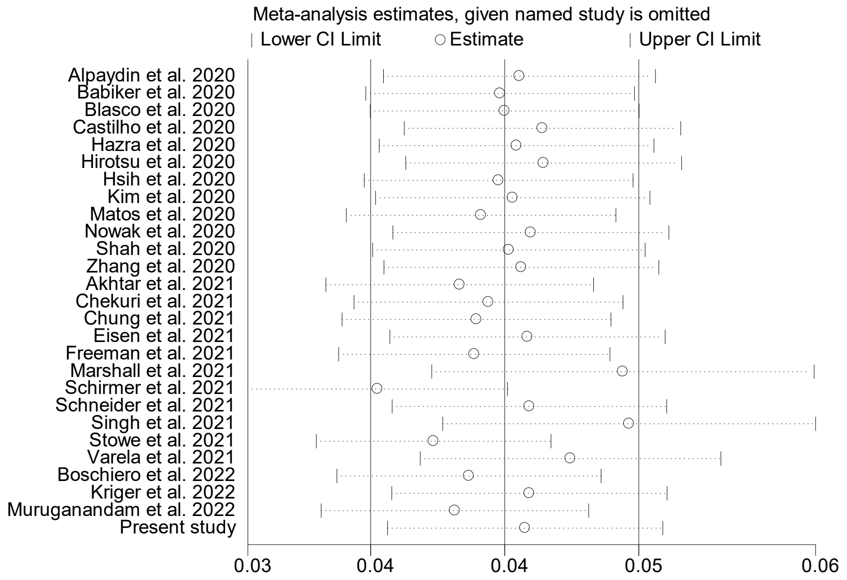

Figure S9. Sensitivity analysis for the effect of removing one study at a time on the overall estimation of FluAV positivity in SCNG.

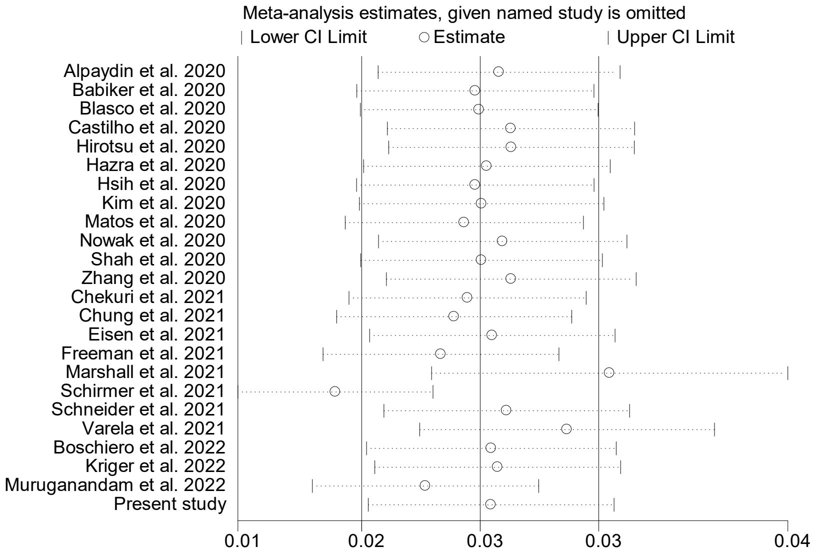

Figure S10. Sensitivity analysis for the effect of removing one study at a time on the overall estimation of FluBV positivity in SCNG.

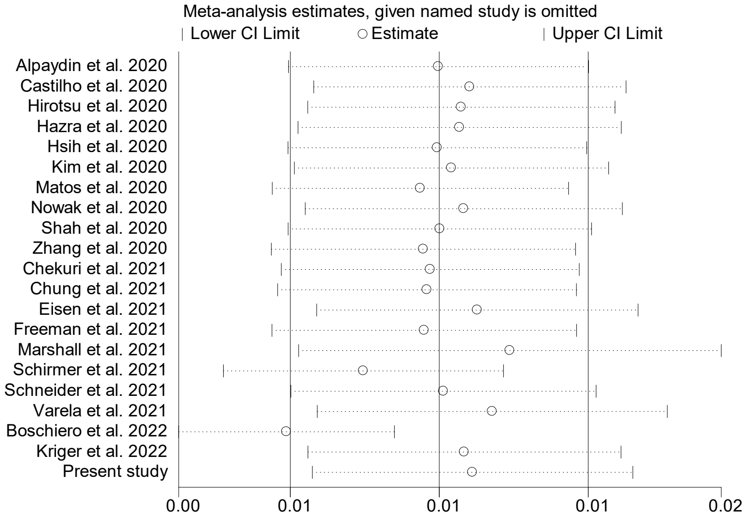

Supplement: Supplementary file 1 [file viruses-15-00665-s001.zip › viruses-2197991-supplementary.pdf]
